# Constitutive Behavior and Finite Element Analysis of FRP Composite and Concrete Members

**DOI:** 10.3390/ma6093978

**Published:** 2013-09-10

**Authors:** Ki Yong Ann, Chang-Geun Cho

**Affiliations:** 1 Department of Civil and Environmental Engineering, Ansan 426, Korea; E-Mail: kann@hanyang.ac.kr; 2 School of Architecture, Chosun University, Gwangju 501, Korea

**Keywords:** FRP and concrete composite, constitutive behavior, compressive test, bending test, mixed finite element

## Abstract

The present study concerns compressive and flexural constitutive models incorporated into an isoparametric beam finite element scheme for fiber reinforced polymer (FRP) and concrete composites, using their multi-axial constitutive behavior. The constitutive behavior of concrete was treated in triaxial stress states as an orthotropic hypoelasticity-based formulation to determine the confinement effect of concrete from a three-dimensional failure surface in triaxial stress states. The constitutive behavior of the FRP composite was formulated from the two-dimensional classical lamination theory. To predict the flexural behavior of circular cross-section with FRP sheet and concrete composite, a layered discretization of cross-sections was incorporated into nonlinear isoparametric beam finite elements. The predicted constitutive behavior was validated by a comparison to available experimental results in the compressive and flexural beam loading test.

## 1. Introduction

As already well known, fiber reinforced polymer (FRP) manufactured by glass or carbon fiber improves concrete properties, in particular, the strength and ductility arising from enhanced confinement of the structural members [1,2,3,4,5,6,7,8,9]. In fact, a significant increase in the concrete properties may be attributed to the confinement of lateral expansion of concrete as being related to its strength and ductility. This achievement is even greater for the triaxial state rather uniaxial or biaxial ones. As fibers in concrete resist the lateral expansion, the concrete member would be subjected to the multi-axial stress state as opposed to the uniaxial stress state. Some computational, mathematical models were previously used to determine the confinement of FRP composite jacket, using the stress-strain curve [6,9,10], covering the shape of the cross-section, the strength, and the material constants in a large range of parametric values. Notwithstanding, no breakthrough for assessing the concrete confinement during loading has been, to date, given, considering a change in the tangent stiffness and lateral expansion, which are strongly affected by the triaxial stress state. 

A confinement model proposed by Mander *et al*. (1988), is often refined to interpret the concrete-filled composites [5,10], as the biaxial strength failure of the FRP composites is taken into account. To determine the compressive and flexural behaviors of FRP-confined concrete, Davol *et al.* (2001) additionally developed a model using an equivalent tangent modulus determined from the total lateral strain [3]. Properties of the FRP composites were substantially achieved by the classical lamination theory together with an iterative model computing the current apparent Poisson’s ratio. Alternatively, an explicit formula may be used to describe the effect of confinements, in particular, as being related to the concrete lateral strain and the axial strain [11].

The present study concerns a development of the nonlinear material model for multi-axial constitutive behaviors to predict the compressive and flexural behaviors of FRP sheet and concrete composites. The constitutive model was developed by the basic concept of an isoparametric beam finite element in the present study. Concrete is regarded as an orthotropic material described by the hypoelasticity-based principle in triaxial constitutive law. Simultaneously, the principal stress direction is assumed to be parallel to the orthotropic directions. In defining the confinement effect, a three dimensional failure surface in the current concrete triaxial stress state was formulated together with behavior of the FRP sheet, being based on the two-dimensional lamination theory for composite laminated materials. To predict the flexural behavior of circular cross-section with FRP sheet and concrete composite, a layered discretization of cross-sections was employed, considering nonlinear isoparametric beam finite elements. After cracking of concrete, the effect of tension stiffening due to the presence of the FRP jacket is also taken into account. Finally, the constitutive model was verified by a comparison to experimental results of compressive and flexural beam loading test.

## 2. Constitutive Model for FRP Composite and Concrete Circular Section

### 2.1. Laminate Composite for FRP Composite

**Figure 1:**
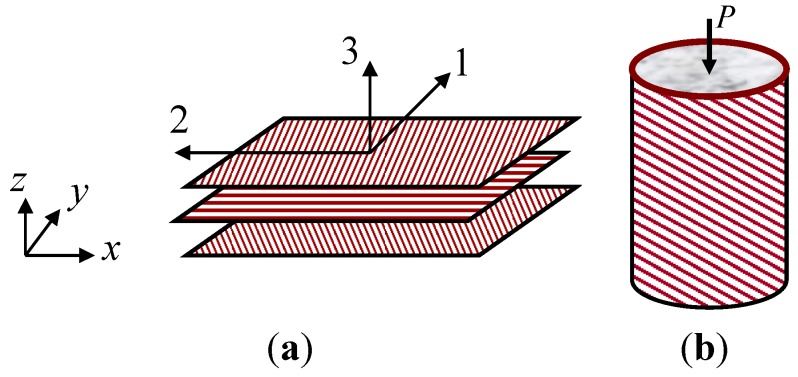
(**a**) fiber reinforced polymer (FRP) laminated composite; and (**b**) FRP jacketed concrete cylinder in compression.

A single lamina of the FRP sheet consists of unidirectional fibers embedded in the matrix, which is taken as a transversely isotropic material in modelling the plane normal to the fiber direction, as seen in Figure 1a. The stress-strain relation for lamina in the local coordinate (*i.e.*, 1, 2 and 3) and in the structure coordinate (*x*, *y* and *z*) can be respectively expressed, as follows:

(1) $$\sigma_{1}=Q\;\varepsilon_{1}$$

(2) $$\sigma =\bar{Q}\;\varepsilon$$

(3) $$\bar{Q}=T^{-1}\;Q\;T^{-T}$$

where ***T*** indicates the transformation matrix; the σ and ε are for the stress and strain vectors in the local coordinate (*x*, *y*, and *z*), respectively; while the σ and ε_1_ are for the stress and strain vectors in the structure coordinate (1, 2, and 3), respectively. Simultaneously, the coefficients of the constitutive matrix ***Q*** can be rendered as follows:

(4) $$Q_{11}=E_{1}/\eta_{12},\;Q_{12}=Q_{21}=E_{2}\nu_{12}/\eta_{12},\;Q_{22}=E_{2}/\eta_{12},\;Q_{33}=G_{12},\;\eta_{12}=1-\nu_{12}\nu_{21}$$

where *E*_1_ is for the modulus of the lamina in the fiber direction; *E*_2_ for the modulus normal to the fiber direction; $\nu_{ij}$ for the Poisson’s ratio for the deformation in the j direction in loading in the i direction; and *G*_12_ for the in-plane shear modulus of the lamina.

From the constitutive equation for a single lamina, the in-plane stress-strain relation of a laminated composite can be an equivalent orthotropic composite material in the coordinate (*x*, *y*, and *z*).

(5) $$A_{ij}=\sum_{k=1}^{n}{\left({\bar{Q}}_{ij}\right)}_{k}\left(h_{k}-h_{k-1}\right)\;i,j=1,..,3$$

where n is for the total number of plies; and $h_{k}$ for the distance from the mid-plane of the lamina to each ply. In applying to concrete structural beam or column members, a combination of the FRP sheet and concrete can refine the innovated strength and deformation capacity of the structure, as seen in Figure 1b.

**Figure 2:**
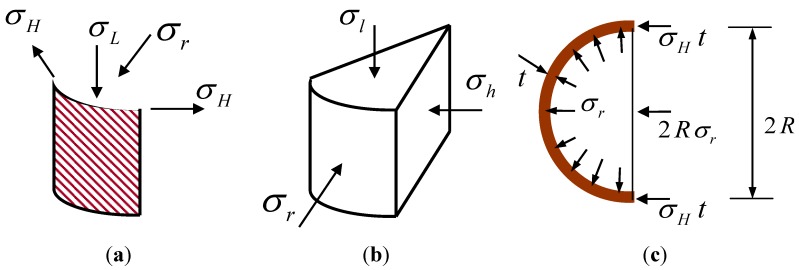
(**a**) In-plane stresses in FRP jacket; (**b**) triaxial stresses in concrete; and (**c**) equilibrium in cross-section.

In Figure 2a, the *x* and *y* axis were set parallel to the longitudinal, hoop direction of cylinder, for which the strain of the FRP laminate composite can be subsequently determined by the laminate symmetric as there is no shear coupling, considering the absence of shear coupling as:

(6) $$\varepsilon_{L}=\frac{\sigma_{L}}{E_{L}}-\nu_{HL}\frac{\sigma_{H}}{E_{H}},\;\varepsilon_{H}=\frac{\sigma_{H}}{E_{H}}-\nu_{LH}\frac{\sigma_{L}}{E_{L}}$$

where *E_L_* and *E*_H_ are for the elastic modulus of the longitudinal and hoop direction, respectively; and ν*_LH_* for the Poisson’s ratio of the FRP laminate composite.

(7) $$E_{L}=\frac{A_{11}A_{22}-A_{12}^{2}}{tA_{22}},\;E_{H}=\frac{A_{11}A_{22}-A_{12}^{2}}{tA_{11}},\;\nu_{LH}=\frac{A_{12}}{A_{22}},\;\nu_{HL}=\frac{A_{12}}{A_{11}}$$

The subscripts *L* and *H* indicate the longitudinal and hoop directions, respectively; *E_L_* and *E*_H_ are the elastic moduli in the longitudinal and hoop directions, respectively; while ν*_HL_* and ν*_LH_* for the Poisson’s ratios, respectively; and t for the thickness of the laminate.

### 2.2. Stresses and Strains of FRP Composite and Concrete Circular Section

The compressive strength and ductility of concrete depend on confinement degree of the FRP sheet. The multiaxial strain-stress relation of concrete can be expressed as seen in Figure 2b, based on an orthotropic hypoelastic formulation, assuming that concrete properties in the radial are identical to those in the hoop direction [12].

(8) $$\left\{\begin{array}{c} d\sigma_{l}\\ d\sigma_{r} \end{array}\right\}=\frac{1}{\Omega }\left[\begin{array}{cc} E_{l}\left(1-\mu_{rh}^{2}\right) & 2\mu_{lr}\sqrt{E_{l}E_{r}}\left(1+\mu_{rh}\right)\\ \mu_{lr}\sqrt{E_{l}E_{r}}\left(1+\mu_{rh}\right) & E_{r}\left(1-\mu_{rh}\right) \end{array}\right]\left\{\begin{array}{c} d\varepsilon_{l}\\ d\varepsilon_{r} \end{array}\right\}$$

(9) $$\Omega =1-2\mu_{lr}^{2}\left(1+\mu_{rh}\right)-\mu_{rh}^{2}$$

where *E_l_* and *E_r_* are the concrete tangent moduli in the axial and radial directions, respectively; and *μ_ij_* is derived from the Poisson’s ratios ν*_ij_* as

(10) $$\mu_{lr}^{2}=\nu_{lr}\nu_{rl},\;\;\mu_{rh}^{2}=\nu_{rh}\nu_{hr},\;\;\mu_{lh}^{2}=\nu_{lh}\nu_{hl}$$

The constitutive equation can be transformed by the equivalent uniaxial strain concept into the two independent uniaxial constitutive relations [13].

(11) $$d\sigma_{i}=E_{i}d\varepsilon_{iu}$$

where ε*_iu_* is the concrete equivalent uniaxial strain. The material in the strain in direction i would exhibit if subjected to a uniaxial stress σ*_i_* with other stresses equal to zero, and derived as follows

(12) $$d\varepsilon_{iu}=\left[\left(1-\mu_{rh}^{2}\right)d\varepsilon_{l}+2\mu_{lr}\sqrt{E_{r}/E_{l}}\left(1+\mu_{rh}\right)d\varepsilon_{r}\right]/\Omega$$

The compatibility in the cross-section should be satisfied as shown in Figure 2c, assuming that the interfaces between the concrete and the FRP sheet are perfectly bonded. From the Equation (6) with the compatibility and equilibrium in the cross-section, the increment of radial stress in concrete, dσ*_r_*; the increment of longitudinal stress in the FRP composite, dσ*_L_*; and the increment of radial strain in concrete, dε*_r_*;, can be derived, respectively

(13) $$d\sigma_{r}=-\frac{\left(\nu_{LH}\;d\varepsilon_{l}+d\varepsilon_{r}\right)E_{H}t}{\left(1-\nu_{HL}\nu_{LH}\right)R},\;d\sigma_{L}=E_{L}\left(d\varepsilon_{L}-d\sigma_{r}\frac{\nu_{HL}R}{E_{H}t}\right)$$

(14) $$d\varepsilon_{r}=d\varepsilon_{l}\left[\frac{\nu_{LH}E_{H}t\left(1-\mu_{rh}-2\mu_{lr}^{2}\right)+\mu_{lr}\sqrt{E_{l}E_{r}}R\left(1-\nu_{HL}\nu_{LH}\right)}{-E_{r}R\left(1-\nu_{HL}\nu_{LH}\right)-E_{H}t\left(1-\mu_{rh}-2\mu_{lr}^{2}\right)}\right]$$

where R is the radius of the concrete core; and t for the total thickness of the FRP composite.

### 2.3. Constitutive Model of Concrete in Triaxial Stress State

**Figure 3:**
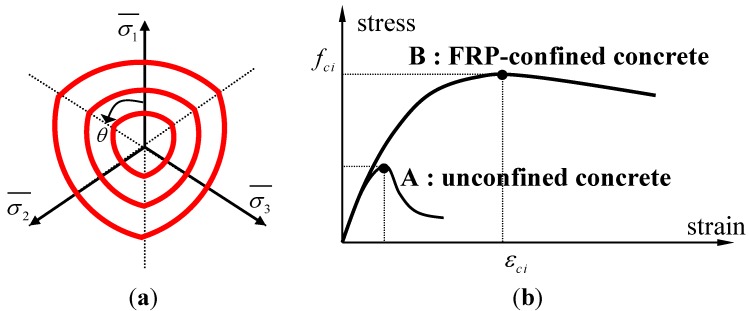
(**a**) Five-parameter failure surface of concrete; and (**b**) confined and unconfined stress-strain curve of concrete in compression.

As the behavior of concrete wrapped by a FRP jacket is triaxial rather than uniaxial, a triaxial constitutive model is required. To determine the tri-axial strength of confined concrete wrapped by FRP composite, as shown in Figure 3a, the tri-axial failure surface of concrete proposed by a five-parameter model [14], is adopted to the stress state defined as σ*_r_* and σ*_r_* = σ*_h_*. In the test of confined concrete, it is known that the radial and hoop stresses of concrete were mostly identical and thus the assumption has been commonly adopted in the majority of previous studies [2,3,10].

A strain surface is required to define the peak compressive strain, *ε'_ci_* in the equivalent uniaxial stress and strain curve of concrete as shown in Figure 3b. Concrete becomes more ductile when the level of confinement is increased. In the present research, the strain surface of concrete wrapped by FRP composite is defined as the equation of strength enhancement factor [1,15]. To model the equivalent uniaxial stress-strain relation of the unconfined and confined concrete is shown in Figure 3b, Saenz’s curve is adopted to describe the equivalent uniaxial compressive stress-strain curve of concrete [16]. On the other hand, the Poisson’s ratio of concrete was adopted as a cubic function that predicted the volume expansion observed during experimental studies of concrete and this model was well matched with the experimental results [12]. Hence, after reaching the failure strength of the FRP composite in a section, it is assumed that the member does not resist additional loads and finally fails. In the proposed model, the Tsai-Wu failure criterion was applied to predict the failure of the FRP composite [17]. Tsai and Wu [17] postulated a general form of a failure surface in six-dimensional stress space. For the case of the in-plane composite behavior as an FRP jacket, the criterion could be minimized to have three parameters of a composite material; two axial strengths both in 1- and 2-directions and one shear strength. 

## 3. Mixed Beam Finite Element with Layered FRP Concrete Composite Section

**Figure 4:**
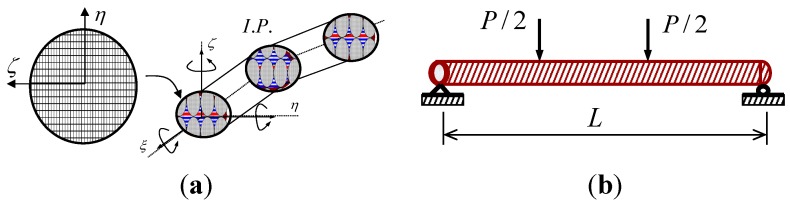
(**a**) Layered isoparametric beam finite element; and (**b**) four-point bending test of FRP and concrete circular beam.

The layered cross-sectional discretization, as shown in Figure 4a, was incorporated into an isoparametric beam finite element by the Hellinger-Reissner type mixed formulation [18]. This formulation is a two-field variational method derived by combining the compatibility conditions with the principle of virtual work. 

### 3.1. Finite Element Formulation

The weak form of the compatibility conditions can be derived by applying a statically admissible virtual force system as

(15) $$\int_{L}\delta D^{T}\left(d^{i-1}+f^{-1}\Delta D^{i}\right)dx-\int_{L}\delta D^{T}\partial \left(y^{i-1}+\Delta y^{i}\right)dx=0$$

where ***D*** is the section force vector consisting of section moments; ***d****^i^* for the section deformation vector consisted of section curvature; *y^i^* for the displacement fields at the *i*th Newton-Raphson iteration; and ***f*** for the section flexibility matrix, respectively. By the principle of virtual work, the equilibrium can be formulated in relation of the section deformation vector, ***d*** = ∂*y*

(16) $$\int_{L}\delta y^{T}\left(D^{i}+\Delta D^{i-1}\right)dx-\int_{L}\delta y^{T}pdx-\delta q^{T}Q=0$$

where δ***d*** is the virtual section deformation vector caused by the virtual displacement δy; **Q** and *q* for the nodal force and nodal displacement vectors, respectively; while *p* for the applied load vector per unit length. The Hellinger-Reissner mixed formulation can be derived by a combination of the compatibility and equilibrium equations as:

(17) $$\int_{L}{\left\{\begin{array}{c} \delta D\\ \delta y \end{array}\right\}}^{T}\left[\begin{array}{cc} -f^{-1} & \partial \\ \partial^{T} & 0 \end{array}\right]\left\{\begin{array}{c} \Delta D^{i}\\ \Delta y^{i} \end{array}\right\}dx=\int_{L}{\left\{\begin{array}{c} \delta D\\ \delta y \end{array}\right\}}^{T}\left\{\begin{array}{c} 0\\ p \end{array}\right\}dx+\delta q^{T}Q+\int_{L}{\left\{\begin{array}{c} \delta D\\ \delta y \end{array}\right\}}^{T}\left\{\begin{array}{c} d^{i-1}-\partial y^{i-1}\\ -\partial^{T}D^{i-1} \end{array}\right\}dx$$

To make it feasible the variational expression of equilibrium relation in a finite element approach, it must be discretized in terms of nodal quantities. In a mixed finite element formulation, the internal forces ***D*** are interpolated in terms of the force degrees of freedom **Q**. Also the displacement fields *y* are interpolated in terms of the nodal displacements *q*.

By applying the interpolation of internal forces and displacement fields, the function of the mixed finite element formulation can be obtained as

(18) $$K^{i-1}\Delta q^{i}=P_{e}+Q-Q^{i-1}+k_{qQ}^{i-1}{\left(k_{QQ}^{i-1}\right)}^{-1}q_{r}^{i-1}$$

where ***K****^i^*^−1^ is the element stiffness matrix at the end of the last iteration defined as follows:

(19) $$K^{i-1}=-k_{qQ}^{i-1}{\left(k_{QQ}^{i-1}\right)}^{-1}k_{Qq}^{i-1}$$

(20) $$k_{QQ}^{i-1}=\int_{L}-b^{T}f^{-1}b\;dx\text{, }k_{qQ}^{i-1}=\int_{L}{\left(\partial a\right)}^{T}b\;dx\text{, }k_{Qq}^{i-1}=\int_{L}b^{T}\partial b\;dx$$

where *b* and *a* are the interpolation functions for the internal forces and the element displacements, respectively; while *P_e_* and *q_r_^i^*^−1^ for the equivalent nodal load and nodal residual vectors, respectively;

(21) $$P_{e}=\int_{L}a^{T}pdx\text{, }q_{r}^{i-1}=-\int_{L}b^{T}\left(d^{i-1}-\partial y^{i-1}\right)dx$$

and, ***Q****_r_^i^*^−1^ is the element resisting forces defined as

(22) $$Q_{r}^{i-1}=-\int_{L}{\left(\partial a\right)}^{T}D^{i-1}dx$$

### 3.2. Layered Section Discretization

To compute the sectional force vector and flexibility matrix, corresponding to the section deformation in the element formulation, each cross-section relies on the layered cross-sectional discretization as seen in Figure 4a. The section force-deformation relation is determined by integration of the stress-strain behavior of FRP and concrete composite layers as expressed in the previous section. For concrete in tension, it was assumed that the response is linearly elastic in the pre-cracked region but the tensile stress of concrete becomes zero after reaching the tensile strength of concrete. Since the section moment and force are obtained by integrating all layered stresses, the moment and axial force are totally coupled.

## 4. Analysis of FRP and Concrete Composite Cylinder in Compression 

A comparison of the available experimental tests to the analysis result, predicted by the current model in chapter 2, was performed. The two series of experimental tests involving axially loaded FRP and concrete composite cylinders were investigated in this section. 

**Figure 5:**
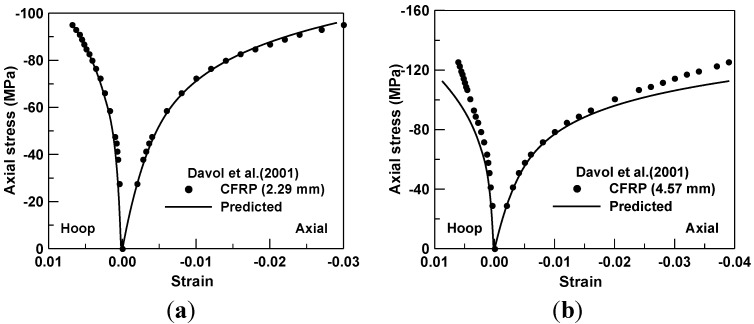
(**a**) Prediction of compression test for CFRP thickness of 2.29 mm; and (**b**) prediction of compression test for CFRP thickness of 4.57 mm.

A compression test for the CFRP and concrete composite cylinder was taken with a cylindrical specimen of 152.0 mm diameter [3], as shown in Figure 1b. Two composite architectures were used for the CFRP composite sheets to confine the concrete. For two specimens, the total thickness of the FRP sheet was 2.29 mm and 4.57 mm, respectively and the lay-ups of CFRP composite were designed so that 85% of the fibers were placed at ±10° in the angle with 15% remaining placed at ±90° with respect to the longitudinal axis of the cylinder. For the lamina of CFRP sheet, the longitudinal and transverse elastic modulus was 121 GPa and 6.90 GPa, respectively. The shear modulus was 4.83 GPa, and the Poisson’s ratio was 0.3. The tensile and compressive strength in the fiber direction of the FRP composite was 966 MPa. The tensile strength normal to the fiber direction is 42 MPa. The in-plane shear strength was 95 MPa. The unconfined compressive strength of the concrete was 45.5 MPa and the corresponding strain was 0.003. The prediction for the stress and strain behavior with 2.29 mm of the CFRP thickness, as shown in Figure 5a, was mostly equated to the experimental response from the initial to the ultimate loading stage. However, minor discrepancies were observed as shown in Figure 5b, in the specimen of the CFRP with 4.57 mm of the thickness, when the compressive load was reaching the maximum load.

**Figure 6:**
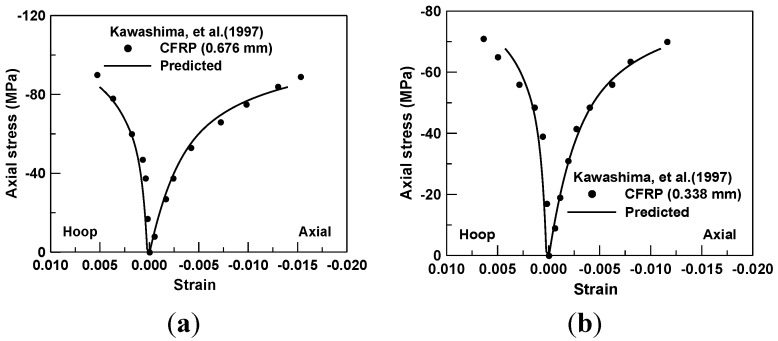
(**a**) Prediction of compression test for CFRP thickness of 0.676 mm; and (**b**) prediction of compression test for CFRP thickness of 0.338 mm.

The compression test of CFRP and concrete cylinders with 200 mm in the diameter and 600 mm in the height was also confirmed by the prediction obtained from the proposed model [7]. Two specimens were manufactured with the total thickness of CFRP sheet (*i.e.*, 0.338 mm and 0.676 mm, respectively). The fiber tensile strengths of the two specimens accounted for 2810 MPa and 2327 MPa, respectively. Simultaneously, the elastic modulus of the CFRP layer was 439 MPa along the fiber direction, while the uniaxial unconfined concrete strength was 39 MPa. For the specimen of the CFRP thickness of 0.676 mm, as shown in Figure 6a, the axial and hoop strains were very identical with the tested responses. The predicted results for the specimen with the FRP with the thickness of 0.338 mm indicated more or less the discrepancy around the failure point in the hoop strain; however, the overall predicted results showed a good agreement with the tested behaviors.

## 5. Finite Element Prediction of FRP and Concrete Composite Beams

**Figure 7:**
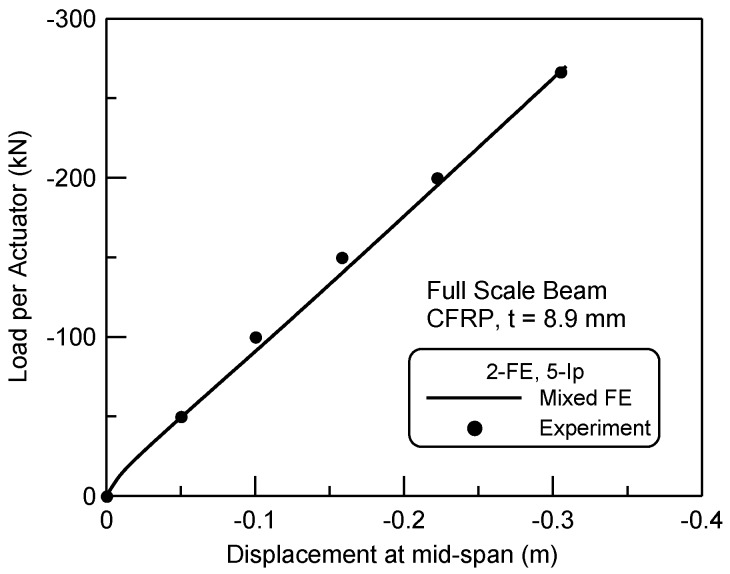
Transverse load and displacement responses of the beam.

The four-point bending test of a CFRP and concrete composite beam [2] was taken, as shown in Figure 4b, to compare to the mixed finite element prediction. The composite beam was loaded transversely with the span of 7.93 m in the length and the shear-span of 2.74 m in the length, while the circular cross-section was designed to be 343 mm in the diameter. The total thickness of the FRP sheet wrapping the concrete was 8.9 mm and the lay-ups of the sheet were designed so that 68.6% of the fibers were placed at ±10° in the angle with 31.4% of the remaining placed at ±90° with respect to the longitudinal direction of the beam. The properties of the CFRP composite were equated to the compressive test as described in the previous chapter [3]. The unconfined compressive strength of the concrete was 20.7 MPa, while 0.003 for the corresponding strain in compression, and 15.4 GPa for the initial elastic modulus of concrete. For the symmetric condition of the four-point bending beam test about the center span, only half of the beam in the left was modeled by two finite elements with five integration points per an element. From the nonlinear mixed finite element analysis, the predicted transverse load and mid-span displacement response was presented to compare to the experimental result as shown in Figure 7. The predicted load-displacement curve well accorded with the experiment both in the initial load level and in the ultimate load level. The ultimate load measured in the experiment was 272.3 kN, whilst 272.6 kN was predicted in the finite element analysis, imposing that the error was only +0.07%.

**Figure 8:**
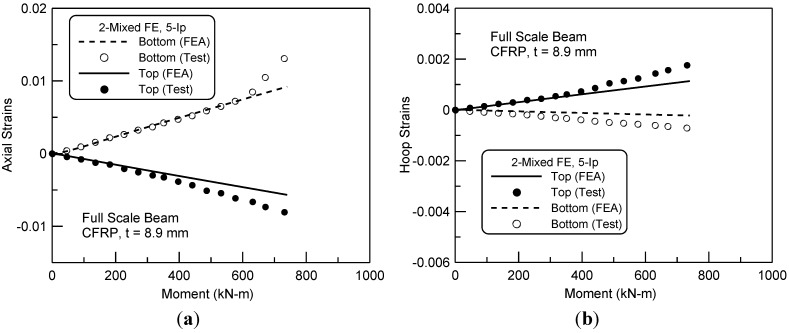
(**a**) Axial strains and bending moment relationship at center span; and (**b**) hoop strains and bending moment relationship at center span.

The axial and hoop strains of the top and bottom fiber of the cross-section in the center span were presented in Figure 8. The predicted axial strains both in the extreme compressive and tensile fibers well accorded with the experimental result. However, the discrepancy of axial strains was observed in near the ultimate load level. This was again observed in the prediction of hoop strains as shown in Figure 8b. Substantially, the finite element analysis underestimated the axial and hoops strains in ultimate, as being confirmed with the experimental result. It may be attributed to the expansion of the concrete in compression and thus the propagation of crack width in tension at around the ultimate load level. The current macro-level model of the hypo-elasticity material formulation could not unfortunately predict precisely the extremely expansion of crushing and crack width of concrete. Moreover, the nonlinear bond mechanism is not clear in the layers between the FRP sheet and the expanded concrete at ultimate to date.

## 6. Conclusions

The compressive and flexural constitutive behavior of a FRP sheet and concrete composite was formulated from their multiaxial constitutive laws and the material constitutive models, incorporating into an isoparametric mixed beam finite element scheme with the circular cross-section of the beam. The nonlinear local cross-sectional strains both in axial and lateral directions was predicted by the model developed in the present study, and then the confining behavior of concrete was rationally estimated when wrapped by FRP composite sheet.

In a comparison of the analysis to the experiment on the compressive cylinder test and bending load test to beams, the compressive behavior of CFRP sheet and concrete composite cylinder was well predicted. Also the overall transverse load and displacement response of the beam indicated a positive consistent result. Additionally, a comparison of the model for the axial and hoop responses in CFRP sheet and concrete composite sections to the experiment was taken to estimate the expansion of crushed concrete at ultimate load level.
